# Non-Contact Surface Roughness Measurement by Implementation of a Spatial Light Modulator

**DOI:** 10.3390/s17030596

**Published:** 2017-03-15

**Authors:** Laura Aulbach, Félix Salazar Bloise, Min Lu, Alexander W. Koch

**Affiliations:** 1 Institute for Measurement Systems and Sensor Technology, Technische Universität München, Theresienstrasse 90, 80333 Munich, Germany; min.lu@tum.de (M.L.); a.w.koch@tum.de (A.W.K.); 2 ETSI Minas y Energía, Universidad Politécnica de Madrid, Rios Rosas 21, 28003 Madrid, Spain; felixjose.salazar@upm.es

**Keywords:** roughness measurement, fringe visibility, non-contact method, spatial light modulator, liquid crystal device

## Abstract

The surface structure, especially the roughness, has a significant influence on numerous parameters, such as friction and wear, and therefore estimates the quality of technical systems. In the last decades, a broad variety of surface roughness measurement methods were developed. A destructive measurement procedure or the lack of feasibility of online monitoring are the crucial drawbacks of most of these methods. This article proposes a new non-contact method for measuring the surface roughness that is straightforward to implement and easy to extend to online monitoring processes. The key element is a liquid-crystal-based spatial light modulator, integrated in an interferometric setup. By varying the imprinted phase of the modulator, a correlation between the imprinted phase and the fringe visibility of an interferogram is measured, and the surface roughness can be derived. This paper presents the theoretical approach of the method and first simulation and experimental results for a set of surface roughnesses. The experimental results are compared with values obtained by an atomic force microscope and a stylus profiler.

## 1. Introduction

The roughness of surfaces is a key parameter in a broad variety of technological systems. Just to name a few: in the area of aeronautical engineering, the surface roughness influences the flight dynamics and the wear due to friction [1]; in optical coating and layer processing, the substrate roughness affects the quality of applied layers [2,3]; in the pharmaceutical industry, the quality and effect of tablets can be analysed by the surface roughness [4]; and the quality of optical systems is also related to the roughness of the implemented components [5,6]. Thus, accurate and reliable methods for measuring the surface roughness are in high demand.

Generally, surface roughness measurement techniques can be divided into contact and non-contact types. The first group, including stylus profilers or atomic force microscopes, may lead to damage during the measurement procedure, caused by contact stress between stylus and surface. Non-contact methods are non-destructive and usually optical. The information about the surface roughness is imprinted on an optical wave, which makes the measured object highly accessible and is therefore applicable in online quality monitoring. On the other hand, the non-contact methods are less reliable and reproducible.

Furthermore, the measurement of surface roughness can be divided into (i) profiling methods, which measure the roughness along a specified sampling line Z(x), e.g., stylus or optical profilers [7,8,9]; (ii) topographical methods, which measure the complete surface function Z(x,y), e.g., white light interferometry [10] or fringe projection [11]; and (iii) surface integrating methods, which deduce the roughness of the surface from a correlating parameter, e.g., speckle correlation [12,13] or light scattering methods [14]. Profiling methods are comparatively fast but restricted to random measurements. Topographical methods can capture the complete roughness distribution and are usually interlinked with scanning procedures, which leads to high measurement and pre-adjustment effort. For type (iii) methods, the roughness is measured independently of the location (i.e., a mean value of the roughness is estimated and cannot be assigned to a certain surface area) [15].

This study presents a novel non-contact surface roughness measurement method that combines the advantages of type (i)–(iii) methods, using a Michelson setup with a spatial light modulator (SLM) as reference. SLMs are optoelectrical or optomechanical devices for a local manipulation of the amplitude or phase (or both). Due to the rising variety of commercial SLMs in the last decade, adaptive optical systems have attracted much attention in the field of surface analysis. Most common are micromirror arrays and liquid-crystal-based spatial light modulators (LCSLMs). Previous studies describe the application of SLMs for the measurement of vibrations [16] or contour [17] of optical rough surfaces. Now, the optical sensing by means of a SLM will be applied at the nanoscale for the measurement of surface roughnesses.

It is well known that when a wave, after interacting with a rough surface, interferes with a tilted, plane reference wave to generate a fringe pattern, the resulting fringe visibility is affected by the surface roughness [18,19,20]. If the reference and the object are both rough, the fringe visibility of the resulting interferogram is related to the surface roughnesses or surface height variations $Z_{o}(x,y)$ and $Z_{r}(x,y)$ of object and reference. Modulating the reference roughness by the SLM leads to a functional relation between the fringe visibility and the surface roughnesses, from which the object roughness can be derived. In this article, the theoretical approach of the method is presented and first simulated and experimental results are discussed.

## 2. Theoretical Approach

The principle of the presented method is based on the two-beam interference of complex fields. For the theoretical approach, the relation between the averaged fringe visibility and the respective roughness of object and reference will be discussed without regard to the exact experimental setup. First of all, we assume that the object and the reference are rough surfaces that act as scatterers and introduce pure phase modulation into the complex field. The amplitudes are constant and distributed uniformly. Consequently, the complex field U(x,y) of the object and the reference wave can be expressed as

(1) $$U_{o}(x,y)=A_{o}\cdot \exp\left[{\mathrm{iku}}_{o}Z_{o}(x,y)\right]$$

(2) $$U_{r}(x,y)=A_{r}\cdot \exp\left[{\mathrm{iku}}_{r}\left(Z_{r}(x,y)+f_{r}(x,y)\right)\right],$$

where $A_{o,r}$ denotes the complex amplitudes, $u_{o,r}$ the geometrical factors specifying the scatterer [20], $Z_{o,r}(x,y)$ the surface height variations, and k=2π/λ is the wavenumber of the interacting light of wavelength λ. The term $f_{r}(x,y)$ describes the low-frequency part of the reference wavefront that induces the fringes into the interferogram, for example, tilting the reference object about the x- or y-axis generates parallel, equally spaced fringes. The total complex field arising from the two interacting waves is

(3) $$U_{t}(x,y)=U_{o}(x,y)+U_{r}(x,y)=A_{o}\cdot \exp\left[{\mathrm{iku}}_{o}Z_{o}(x,y)\right]+A_{r}\cdot \exp\left[{\mathrm{iku}}_{r}\left(Z_{r}(x,y)+f_{r}(x,y)\right)\right],$$

with a total intensity $I_{t}(x,y)$ of

(4) $$I_{t}(x,y)=|U_{t}(x,y){|}^{2}=A_{o}^{2}+A_{r}^{2}+A_{o}\cdot A_{r}\cdot \left\{\begin{array}{l} \exp\left[{\mathrm{iku}}_{o}Z_{o}(x,y)\right]\cdot \exp\left[-{\mathrm{iku}}_{r}\left(Z_{r}(x,y)+f_{r}(x,y)\right)\right]\\ +\exp\left[-{\mathrm{iku}}_{o}Z_{o}(x,y)\right]\cdot \exp\left[{\mathrm{iku}}_{r}\left(Z_{r}(x,y)+f_{r}(x,y)\right)\right] \end{array}\right\}.$$

We assume that the surface heights $Z_{o}(x,y)$ and $Z_{r}(x,y)$ are normally distributed (i.e., they have first-order probability density functions).

(5) $$p\left(Z_{o,r}(x,y)\right)=\frac{1}{\sqrt{2\pi \sigma_{o,r}}}\exp\left(-\frac{Z_{o,r}^{2}(x,y)}{2\sigma_{o,r}^{2}}\right)$$

The standard deviation $\sigma_{o,r}$ of a surface height, as can be seen in Equation (5), is by definition equal to the root mean square (RMS) roughness $R_{q}$. Thus, from now on, $\sigma_{o,r}$ will be denoted as $R_{q,o,r}$. Based on Equation (5), a convenient relation between the surface height $Z_{o,r}(x,y)$ and the roughness is given as:

(6) $$\langle \exp[\mathrm{iku}\cdot Z_{o,r}(x,y)]\rangle =\langle \exp[-\mathrm{iku}\cdot Z_{o,r}(x,y)]\rangle =\exp\left[\frac{-{\left(\mathrm{ku}R_{q,o,r}\right)}^{2}}{2}\right].$$

So far, the discussed approach is utterly theoretical. If we wish to transfer the model to an experimental setup, the only available parameter is the averaged intensity of the total complex field. Substituting Equation (6) into Equation (4) leads to the following expression:

(7) $$\begin{array}{cc} \langle I_{t}(x,y)\rangle & =A_{o}^{2}+A_{r}^{2}+A_{o}\cdot A_{r}\cdot \left\{\begin{array}{l} \exp\left[\frac{-{\left(ku_{o}R_{q,o}\right)}^{2}}{2}\right]\cdot \exp\left[\frac{-{\left(ku_{r}R_{q,r}\right)}^{2}}{2}\right]\\ \cdot \left[\exp\left(-iku_{r}\cdot f_{r}(x,y)\right)+\exp\left(iku_{r}\cdot f_{r}(x,y)\right)\right] \end{array}\right\}\\ & =A_{o}^{2}+A_{r}^{2}+2\cdot A_{o}\cdot A_{r}\cdot \exp\left[\frac{-k^{2}(u_{o}^{2}R_{q,o}^{2}+u_{r}^{2}R_{q,r}^{2})}{2}\right]\cos\left(ku_{r}\cdot f_{r}(x,y)\right). \end{array}$$

The fringe visibility of the formed interference fringes can then be calculated as:

(8) $$\langle V\rangle =\frac{{\langle I_{t}(x,y)\rangle }_{\max}-{\langle I_{t}(x,y)\rangle }_{\min}}{{\langle I_{t}(x,y)\rangle }_{\max}+{\langle I_{t}(x,y)\rangle }_{\min}}=\frac{2\cdot A_{o}\cdot A_{r}\cdot \exp\left[\frac{-k^{2}(u_{o}^{2}R_{q,o}^{2}+u_{r}^{2}R_{q,r}^{2})}{2}\right]}{A_{o}^{2}+A_{r}^{2}}$$

In order to obtain comparable results for the standard deviation $\sigma_{o}$ of the object height, normalising the fringe visibility 〈V〉 is mandatory. For this purpose, a reference fringe visibility $V_{\mathrm{norm}}$ must be determined by replacing both scatters by smooth surfaces with otherwise identical specifications:

(9) $$V_{\mathrm{norm}}=\frac{2\cdot A_{o}\cdot A_{r}}{A_{o}^{2}+A_{r}^{2}}$$

Replacing *k* leads to the following relationship between the normalised averaged fringe intensity $\langle V_{0}\rangle$ and the object roughness $R_{o}$:

(10) $$\langle V_{0}\rangle =\frac{\langle V\rangle }{V_{\mathrm{norm}}}=\exp\left[\frac{-2\pi^{2}(u_{o}^{2}R_{q,o}^{2}+u_{r}^{2}R_{q,r}^{2})}{\lambda^{2}}\right]$$

**Figure 1:**
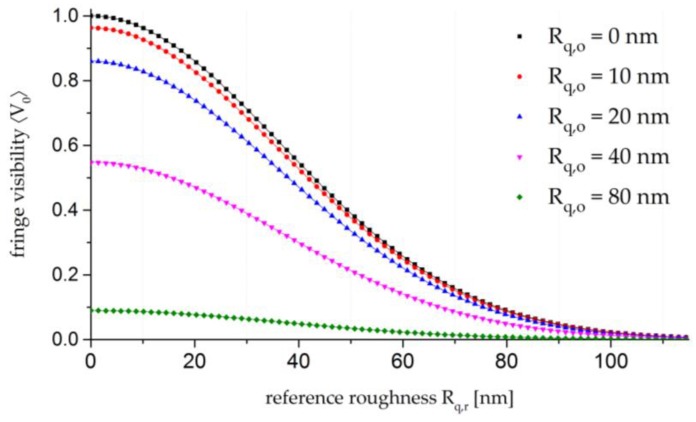
Graphical illustration of Equation (10) for different values of $R_{q,o}$. The curves are calculated for λ = 457.9 nm, $u_{o}$ = $u_{r}$ = 2, and weak scatterers with $R_{q,o}$ and $R_{q,r}<\lambda /4$.

Now that the theory has been described, the limitations of the method will be considered. The estimation of $R_{q}$ is based on the fringe visibility, and therefore the development of fringes is the main precondition. To obtain fringes by interference of the complex amplitudes $U_{o}(x,y)$ and $U_{r}(x,y),$ the phase variations introduced by the rough surfaces must be smaller than 2π in total [21]. This restricts the application of the method to weak scatterers (as object and reference surface) that fulfil the following condition:

(11) $$k\cdot u\cdot R_{q}<\pi \;\mathrm{and}\;k\sum_{n}u_{n}R_{q,n}<2\pi ,$$

where *n* denotes the number of interacting, scattering surfaces. As previously described, the basic idea of the method is to vary the reference roughness $R_{q,r}$ with an LCSLM to derive the object roughness $R_{q,o}$, as illustrated in Figure 1.

A closer evaluation of Equation (10) shows that the object roughness $R_{q,o}$ is proportional to the peak value of the curve plotted in Figure 1 and can be analysed by means of an exponential best-fit curve. In comparison to measurements with a smooth reference surface [18], which are based on a single-point evaluation of the object roughness $R_{q,o},$ this novel approach leads to a higher accuracy due to the best-fit curve.

In comparison with the most commonly used roughness parameter $R_{a}$, which describes the arithmetic or centre-line average of the surface height, the $R_{q}$ roughness is slightly more sensitive to peaks and valleys in the surface height Z(x,y), and therefore $R_{q}>R_{a}.$ More details about the relation between $R_{q}$ and $R_{a}$ are given in [15,22].

## 3. Materials and Methods

Before giving a detailed description of the applied measurement setup and models, it is convenient to make a few assumptions. For the purpose of generality, the structure of the scatterers, defined by $u_{o,r}$, was neglected during the theoretical derivation of the method. The used LCSLM and the measurement objects are reflective, so $u_{o}$ = $u_{r}$ = 2 [20]. Due to the condition expressed by Equation (11), the described technique is valid for $R_{q,o}<\lambda /4.$

### 3.1. Experimental Setup

**Figure 2:**
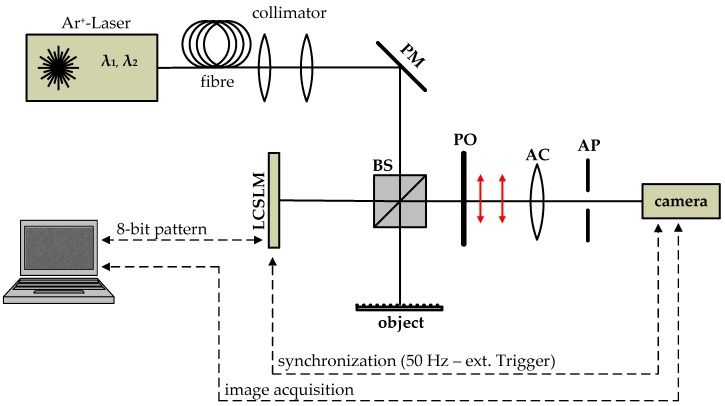
Experimental setup. PM: plane mirror; BS: beam splitter; PO: polariser; AC: achromatic lens; and AP: aperture.

For the measurements and simulations, a classical Michelson setup is deployed, as shown in Figure 2. A collimated illumination beam is divided by a 50/50 beam splitter into an object path and a reference path. The laser source is an Ar^+^-laser head with several laser lines from 457.9 nm to 514.7 nm. For the experiment, the laser head is operated in single line configuration (i.e., for each measurement, a single wavelength is selected). The incident light is reflected by the object and reference surface respectively and interferes after superimposition by the beam splitter.

As reference surface, a reflective liquid-crystal-on-silicon modulator is implemented. The phase imprinted on the LCSLM is dependent on the polarisation and requires a certain polarisation angle for the readout. Hence, the superimposed wave has to pass through a polariser before hitting the detector. The detector is a 1312 × 1082 pixel complementary metal-oxide semiconductor (CMOS) camera with a pixel size of 8 $\mu m^{2}$.

For stable image acquisition, the refresh rate $f_{\mathrm{sync}}=$ 50 Hz of the liquid crystals needs to be synchronised with the camera by an external trigger signal. Therefore, the exposure time $\tau_{\exp}$ of the camera has to fulfil the following condition:

(12) $$\tau_{\exp}=n\cdot \tau_{\mathrm{sync}}\;\mathrm{and}\;\tau_{\mathrm{pattern}}>\tau_{\exp}+\tau_{\mathrm{sync}},$$

where $\tau_{\mathrm{pattern}}$ denotes the time per imprinted phase pattern on the LCSLM, *n* is a positive integer (1,2,3,…), and τsync=1fsync. If this condition is ignored, the imprinted phase pattern changes possibly during camera exposure and the recorded image is inaccurate.

For imaging, an achromatic lens with focal length 80 mm and an adjustable aperture are used. The magnification of the system is set to 1.4, so that an area of 7.5 mm × 6.4 mm is recorded of both surfaces. To generate the fringes by means of the measurement system, the sample is tilted along the y-axis by a few multiples of the wavelength.

### 3.2. Liquid-Crystal-Based Spatial Light Modulator

The principle of the presented technique is to vary the reference surface roughness with a LCSLM. Thus, the LCSLM is the central element of this work and has to be selected carefully. To accurately model the roughnesses with an SLM, three key factors have to be considered:

1. Phase-only modulation
2. Wavelength range
3. Pixelation

In order for the theory to remain valid, a constant intensity and therefore a phase-only modulation is mandatory. Secondly, the maximum phase shift obtained by the LCSLM is highly dependent on the wavelength. As expressed in Equation (11), a weak scatterer introduces on average a maximum phase shift of 2π between two adjacent surface points. Therefore, a modulator with a dynamic range of 2π in the considered wavelength area is sufficient.

**Figure 3:**
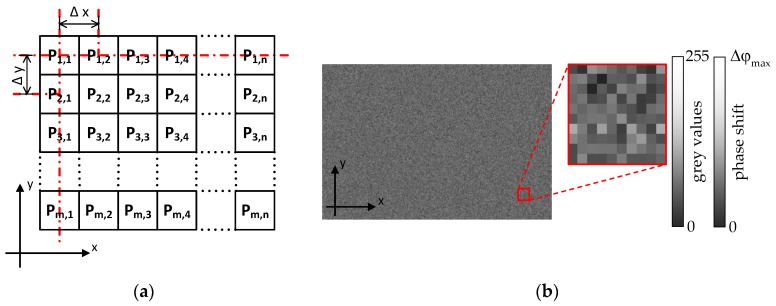
Schematic principle of the phase modulation by the liquid-crystal-based spatial light modulator (LCSLM). (**a**) Pixel structure and (**b**) phase mask corresponding to a roughness of $R_{q}=115\;nm.$

Moreover, a LCSLM with a pixelated modulation structure is required, as schematically shown in Figure 3a. Pixelation means that each pixel element can be addressed separately without influencing the adjacent pixels in any way. The pixelation structure may cause diffraction errors in many fields of use [17,23,24], but for this method this structure is essential, because a continuous modulator structure would lead to a low-pass filtering of the imprinted phase pattern. According to the conditions listed above, the applied modulator is a phase-only LCSLM of type Holoeye PLUTO-VIS with a resolution of 1920 × 1080 pixels. Each pixel is 8 $\mu m^{2}$ in size, and the modulation range is around 4π (for the lines of the Ar^+^-laser: 457.9 nm < λ < 514.5 nm).

The device can easily be addressed as a second computer screen. For operation, the desired phase distribution has to be displayed as an 8-bit grey value pattern on a computer screen. Each grey value represents a certain phase shift of the LCSLM in the range of 0 to $\Delta \varphi_{\max},$ as indicated in Figure 3. By duplicating the grey value pattern of the computer screen to the LCSLM, the modelled phase distribution is introduced pixelwise into the experimental setup. Figure 3b gives an example of a phase pattern that simulates a surface roughness $R_{q}$ of 115 nm.

Due to the non-linear relation between the grey values and the resulting phase shift generated by the LCSLM, calibrating the device to the wavelengths used in practice is mandatory. In this case, the calibration curve is measured by a two-beam interferometer with an implemented double-hole mask. The two beams, generated by the double-hole mask, hit the surface of the LCSLM at two different spots. The phase of one beam is kept constant, while the phase of the second beam is varied by addressing the corresponding area of the LCSLM with the grey values 0–255. This method is recommended by the manufacturer to calibrate the LCSLM and is described in detail in [25]. For higher accuracy, each line was obtained five times and then averaged.

**Figure 4:**
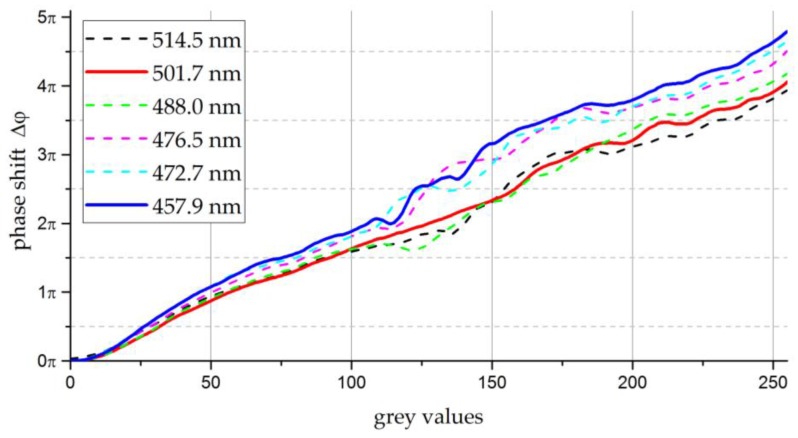
Calibration curves for the Holoeye PLUTO-VIS LCSLM for several lines of an Ar^+^-laser head, obtained by an “18-6” digital addressing sequence.

The Holoeye PLUTO-VIS modulator relies on pulse-width modulation (PWM) to control the liquid crystals. Therefore, the calibration curve can be adjusted by changing the digital addressing sequence of the PWM. In this study, the factory default digital addressing sequence “18-6” is applied. To reduce the phase fluctuations induced by the PWM addressing scheme, Equation (12) must be applied to obtain the calibration curves. The measured calibration curves for several lines of the Ar^+^-laser head are shown in Figure 4.

The two continuous curves in Figure 4 represent the transfer functions of the wavelengths used in the experimental section of this study, λ=457.9 nm and λ=501.7 nm. The dotted curves are recorded for other lines from the Ar^+^-laser head.

Comparing the calibration curves depicted in Figure 4 with curves from literature (e.g., for 633 nm in [26]), the same trend can be observed. Also, the values obtained for the wavelength-dependent modulation range $\Delta \varphi_{\max}$ are in the expected order. Due to the phase fluctuations caused by the digital addressing configuration “18-6”, the calibration curves are noisy. For a proper conversion between the phase and the corresponding grey values, the calibration curves in Figure 4 are shown after smoothing by a Savitzky–Golay filter of window size 5.

### 3.3. Modelling

**Figure 5:**
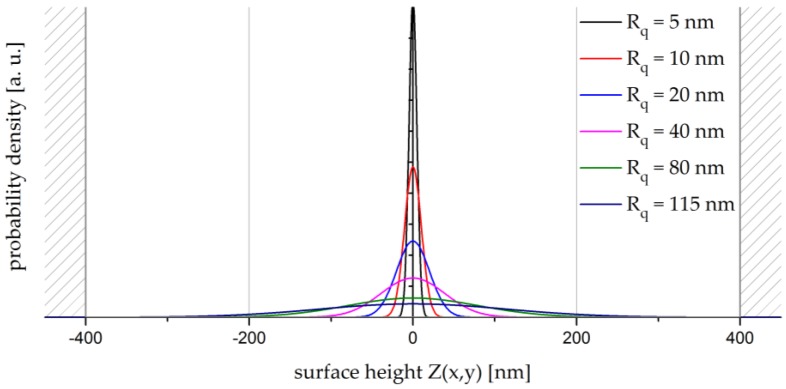
Probability density function of the surface height Z(x,y) for different values of $R_{q}$ in the range 0 < $R_{q}$ < λ/4.

One fundamental aspect of the method is correctly modelling the reference surface roughnesses $R_{q,r}.$ As mentioned above, the roughness RMS $R_{q}$ is equal to the standard deviation σ of the surface height distribution Z(x,y). Figure 5 illustrates the probability density function of Z(x,y) for different values of $R_{q}$.

For small roughnesses $R_{q},$ only low surface height variations with small values for Z(x,y) have to be introduced into the system. For higher roughnesses $R_{q}$, on the other hand, the distribution of Z-values becomes broader. The maximum height difference $Z_{\max}$ realisable by the LCSLM is limited by the modulation range of the device and can be calculated as

(13) $$Z_{\max}=\frac{\Delta \varphi_{\max}}{2\pi }\cdot \lambda ,$$

where $\Delta \varphi_{\max}$ denotes the maximum phase shift (and therefore the modulation range) of the LCSLM.

The LCSLM has a highest modulation range of $\Delta \varphi_{\max}$ = 4.8π at the lowest laser line λ = 457.9 nm, and, inversely, a lowest modulation range of $\Delta \varphi_{\max}$ = 3.9π at λ = 514.5 nm. To prevent error sources, a maximum deflection of the liquid crystals must be avoided so that $\Delta \varphi_{\max}<4\pi$. Based on this condition and Equation (13), $Z_{\max}$ is set to ±400 nm, as represented by the shaded areas in Figure 5. Comparing the boundaries set by $Z_{\max}$ and the broadest probability density function for $R_{q}$ = 115 nm (Figure 5) confirms the feasibility of modelling roughnesses with the LCSLM.

**Figure 6:**
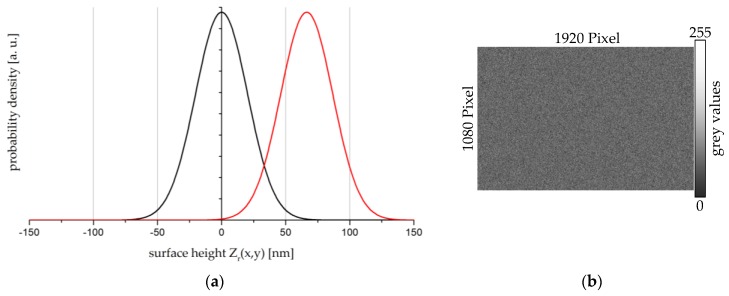
(**a**) Probability density function of the reference surface height $Z_{r}(x,y)$ with standard deviation $R_{q,r}$ = 20 nm. Black curve: centred Gaussian distribution. Red curve: shifted Gaussian distribution; (**b**) grey value pattern for imprinting a certain roughness $R_{q,r}$ on the incident wavefront of the LCSLM.

Finally, the surface function Z(x,y) with standard deviation $R_{q}$ has to be transferred into a grey value pattern in order to operate the LCSLM. The calculation of the grey value pattern can be divided in four steps: (i) Calculate an array (size: 1920 × 1080 pixels) with Gaussian-distributed values for the surface height $Z_{r}(x,y)$. The standard deviation of the Gaussian distribution is equal to the reference roughness $R_{q,r}$ to be modelled. The distribution of $Z_{r}(x,y)$ is shown as an illustrative example for $R_{q,r}$ = 20 nm by the black curve in Figure 6a; (ii) shift the geometrical path lengths $Z_{r}(x,y)$ of the calculated array to a positive range of values (e.g., by red curve in Figure 6a); (iii) transfer the geometrical path lengths $Z_{r}(x,y)$ of the array in phase variations $\Delta \varphi_{r}(x,y)$:

(14) $$\Delta \varphi (x,y)=\frac{Z_{r}(x,y)}{\lambda }\cdot 2\pi .$$

(iv) Transfer the phase variations $\Delta \varphi_{r}(x,y)$ of the calculated array into grey values by considering the calibration curve of the implemented LCSLM. An 8-bit grey value pattern has now finally been generated to operate the LCSLM, as shown in Figure 6b.

### 3.4. Measurement Process and Data Acquisition

**Figure 7:**
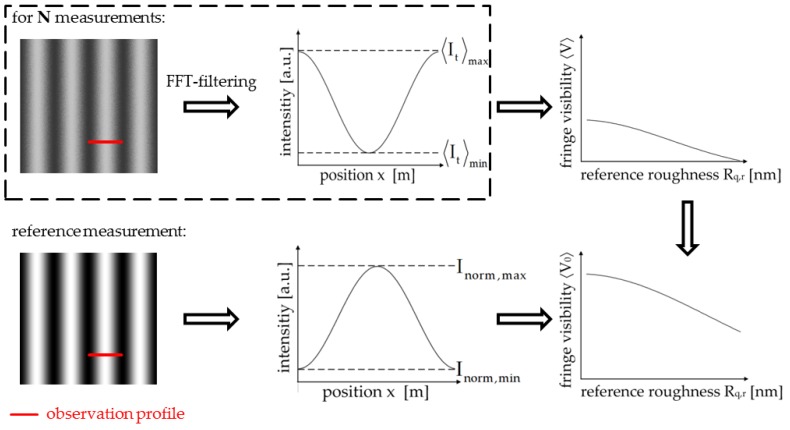
Flow chart for the measurement procedure and data acquisition. N: number of measurements (determined by number of grey value patterns); ${\langle I_{t}\rangle }_{\max,\min}$: extrema of total averaged intensity in a fringe interferogram generated by two rough surfaces with $R_{q,o}$ and $R_{q,r};$ $I_{\mathrm{norm},\max,\min}:$ extrema of intensity in a fringe interferogram generated by two smooth surfaces.

As mentioned above, the presented approach is valid for roughnesses up to λ/4. Therefore, a set of grey value patterns covering the range $\lambda /100<R_{q,r}<\lambda /4$ is calculated (according to step (i)–(iv) described in the preceding section). The grey value patterns are imprinted on the LCSLM consecutively and, for each pattern, a fringe interferogram is recorded. The procedure for retrieving the fringe visibility is illustrated in Figure 7:

At first, the averaged fringe visibility 〈V〉 is calculated for each interferogram, as indicated in the dashed box of Figure 7. Therefore, an observation profile is selected, which has to cover at least one 2π-period of the interferogram. To reduce the noise, the observation profile is filtered in the frequency domain (FFT-filtering). ${\langle I_{t}\rangle }_{\max}$ and ${\langle I_{t}\rangle }_{\min}$ can then be derived, and the fringe visibility for each reference roughness $R_{q,r}$ is calculated according to Equation (8). Secondly, the averaged visibility 〈V〉 has to be normalised for comparison. Therefore, a reference fringe interferogram for two smooth surfaces is generated, as indicated in the bottom interferogram of Figure 7, and a reference fringe visibility $V_{\mathrm{Norm}}$ is deduced.

Finally, the normalised averaged fringe intensity $\langle V_{0}\rangle$ as a function of the reference roughness is derived, according to Equation (10). In the last step, the roughness of the measurement object $R_{q,o}$ is estimated by an exponential regression curve of the form:

(15) $$\langle V_{0}\rangle =\exp\left(a+b\cdot \sigma_{r}^{2}\right)\;\mathrm{with}\;a=\frac{-2\pi^{2}u_{o}^{2}\sigma_{o}^{2}}{\lambda^{2}}\;\mathrm{and}\;b=\frac{-2\pi^{2}u_{r}^{2}}{\lambda^{2}},$$

where *b* is a constant. The roughness of the measurement target $R_{q,o}$ can now easily be determined from the regression parameter *a*.

The presented technique cannot directly be categorised as one of the types (i)–(iii). The fringe visibility is used to deduce the surface roughness, which suggests that the method could be classified as a surface integration method. However, the calculated surface roughness can be allocated to a certain surface area, which is typical of profiling or topographical methods. Here, the calculation of the fringe visibility $\langle V_{0}\rangle$ is based on an observation profile and, therefore, results in a combination of types (i) and (iii). The technique can easily be extended to a topographical method, by performing a pointwise data analysis to generate a 2-dimensional roughness map. To summarise, depending on the selected data analysis process, the method is a mix of either types (i) and (iii) or types (ii) and (iii).

## 4. Results

In order to verify the validity of the new technique, several measurements are carried out. This section presents simulation and experimental results. To find out the relation between the reference roughness $R_{q,r}$ and the fringe visibility, a set of 40 grey value patterns for the LCSLM is calculated according the described procedure. The calibration curve of the LCSLM, which represents the phase shift per grey value, is measured in reflection. Hence, the geometrical factor of the LCSLM now becomes $u_{r}=1$ [20].

To derive the fringe visibility, an observation line of 350 pixels in length is examined. This equals a measuring section of 2 mm on the respective target. The phase response of the LCSLM decreases for higher wavelengths. To prove that the implemented device works over the full available spectral range, the experiments are executed for two lines of the Ar^+^-laser head: 457.9 nm with a maximum phase shift $\Delta \varphi_{\max}=4.4\pi$ and 501.7 nm with $\Delta \varphi_{\max}=3.8\pi$. For the sake of consistency, the mentioned parameters are the same in both the simulation and the experimental approach.

### 4.1. Simulation Results

Prior to experimental testing, the influence of the mask modelling is investigated by simulation. With this aim, two different models for the reference surface height $Z_{r}(x,y)$ are implemented in a simulation environment based on the setup illustrated in Figure 2.

**Figure 8:**
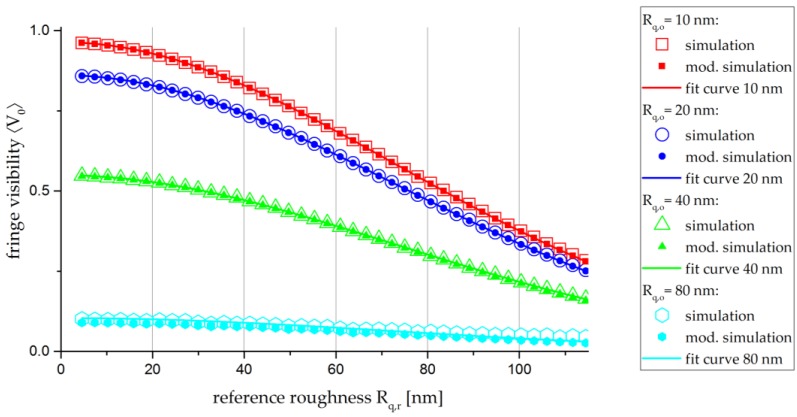
Curve of the fringe visibility $\langle V_{0}\rangle$ as a function of $R_{q,r}$ for different modellings of $Z_{r}(x,y)$ and λ=457.9 nm. Empty dots: standard simulation—Gaussian distribution of $Z_{r}(x,y)$ without modifications; filled dots: modified simulation—shifted and cropped Gaussian distribution of $Z_{r}(x,y)$ . Lines: exponential-fit curves according Equation (15) for the modified simulations.

The first model is a simple Gaussian distribution of the surface height $Z_{r}(x,y)$, as indicated by the black curve in Figure 6. The second model includes the required modifications of the Gaussian distribution for a proper realisation by the LCSLM, that is, the limitation $Z_{r}(x,y)<0.4\;\mu m$ (Figure 5) and the shift into the positive domain (Figure 6—red curve). The simulation results of both modellings of $Z_{r}(x,y)$ and therefore $R_{q,r}$ are depicted in Figure 8. For the standard simulation with the unmodified Gaussian distribution, the curves (filled dots) correspond to the theoretical approach described by Equation (10). The empty dots in Figure 8 represent the fringe visibility $\langle V_{0}\rangle$ as a function of the reference roughness $R_{q,r}$ for the modified Gaussian distribution. The influence of $R_{q,r}$ increases for lower fringe visibilities and, consequently, higher roughnesses $R_{q,o}\;$. Divergences of the modified simulation are visible for $R_{q,o}$ = 80 nm and $R_{q,r}$ > 80 nm.

The values of $R_{q,o}$ and their accuracies are listed in Table 1. $R_{q,o}$ and the respective accuracy are calculated by fitting a curve to the modified simulation values in Figure 8. For the fitting procedure, a Levenberg–Marquardt optimisation algorithm is applied. The obtained data show, as expected, that the simulation result and the calculated simulation input diverge as the object roughness $R_{q,o}$ increases.

The simulation indicates a small divergence from the theoretical approach due to the modification applied to the Gaussian distribution in order to model the reference roughnesses. Nevertheless, the calculated input object roughnesses $R_{q,o}$ are within the accuracy of the fit curve and, therefore, the modelling of the phase masks is valid.

### 4.2. Experimental Results

The suggested method is now applied to three measurement targets with different surface roughnesses. The experimental results are compared with two further widely used techniques—atomic force microscopy (AFM) and stylus profiling.

**Figure 9:**
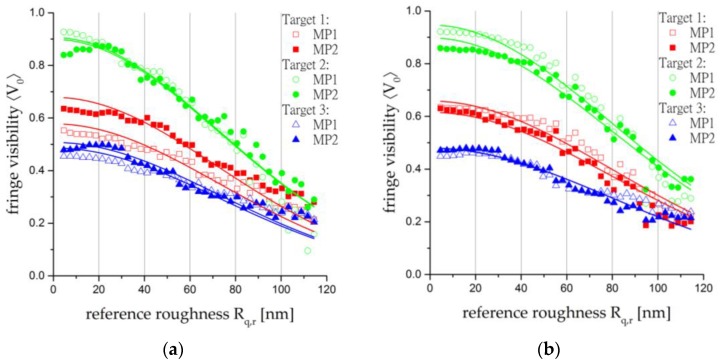
Experimental results and fit curves for the laser lines (**a**) $\lambda_{1}=457.9\;nm$ and (**b**) $\lambda_{2}=501.7\;nm.$

For each target, the roughness is calculated for two different measurement points (MP1 and MP2), i.e., two observation lines per target are evaluated. The overall measurement curves are shown in Figure 9 and the results for $R_{q,o}$ are summarised in Table 2.

In general, it can be said that interpreting surface roughnesses is non-trivial because of a strong dependence on a variety of parameters (e.g., the measurement point and the measurement section). The measurement methods listed in Table 2 are either profiling methods (AFM, stylus profiler) or a mix of profiling and integrating methods (LCSLM), which makes the direct comparison of the values even more complicated. As mentioned previously, the most commonly used roughness parameter is $R_{a}$. If a normal distribution of the surface heights is assumed, the relation $R_{q}\approx 1.25\cdot R_{a}$ can be employed as described in [22]. The experimental results for $R_{q,r}$and the subsequent estimated values of $R_{a,r}$ are shown in Table 2.

On closer inspection of Table 2, the difference between the surface roughnesses measured by AFM and the other methods is notable. This probably arises from the different measurement sections of the methods: the section of AFM—in this article, 2 µm—is much smaller than for the stylus profiler or the LCSLM-based method, which is set to 2 mm, as mentioned above. Hence, AFM is more precise but more sensitive to surface defects such as scratches.

For a full comparison of the methods, the duration of a single surface roughness measurement by each technique is analysed. Of course, the measurement time of a certain technique is highly dependent on several system parameters. For AFM or stylus profiling, the measurement time is limited by the scanning speed of the profiler. The longest time is required by the AFM with 20 min for a 2 µm section. The stylus profiler provides the shortest duration with 10 s for a 2 mm section. For the LCSLM technique, the testing time is related to the number of phase masks and the exposure time $\tau_{\exp}$ of the camera. For a set of 40 phase masks and the shortest possible exposure time (for n = 1 in Equation (16)) a roughness measurement can be completed in 1.6 s. Usually, higher exposure times are necessary (e.g., due to low laser power), and therefore the phase pattern holding time $\tau_{\mathrm{pattern}}$ increases. The duration of a single experiment by the LCSLM presented in this article is around 20 s.

### 4.3. Uncertainty of the Experimental Results

In order to analyse the precision of the proposed technique, the uncertainty of the measurement is estimated. The values shown in Table 2 were obtained by using the parameter *a* in Equation (15), after fitting the exponential curve given in Equation (10). To be certain that the mathematical adjustment was calculated correctly, Cochran´s test for the homogeneity of the dispersions was applied to the $\ln\left(\langle V_{0}\rangle \right)$ as a function of $\sigma_{r}^{2}$ with a significance level of α=0.05 (probability of 95%), and the linearity of the corresponding data was also studied [27]. After obtaining the values of the coefficient *a* for each curve, the random uncertainty was computed by employing the classical theory of errors. Even though the number of samples in each experiment was relatively high, instead of employing the normal or Gauss curve, the Student’s t-distribution was selected for the confidence limits of the parameter *a*.

Taking into account the number of freedom degrees of the measurements, and selecting a confidence interval of 95%, $t_{1-\alpha }=2.025$, approximately. Thus, the confidence interval for a may be expressed as follows:

(16) $$a=\bar{a}\pm U(a)=\bar{a}\pm t_{1-\alpha }\cdot s(a)=\bar{a}\pm t_{0.05}\cdot s(a),$$

where $\bar{a}$ is the value calculated in the fitting corresponding to each specific experiment, s(a) is the standard deviation, and $U(a)=t_{0.05}\cdot s(a)$ represents the uncertainty. Once this uncertainty is known, $U(\sigma_{o}=R_{q,o})$ can be obtained from U(a). The results of the relative uncertainty of $\sigma_{o}$ for the samples of Table 2 are shown in the next table.

All results in Table 3 are below 5%, with the exception of the uncertainty of Target 2 for wavelength $\lambda_{2}$. Analysing all pairs $\left(\ln\langle V_{0}\rangle ,R_{q,r}^{2}\right)$ of the graph depicted in Figure 9b for this experiment (Target 2, MP1) point by point, it can be observed that one of the points is obviously erroneous. That leads to a significant variation in the corresponding standard deviation s(a). In fact, if this value is removed and the same calculation is repeated with (n − 1) values, the uncertainty obtained is 5.4%.

## 5. Discussion

Summing up, the suggested technique is promising. It is a straightforward approach that is easy to implement and the data obtained are broadly consistent with the major trends, as far as comparison is possible. The overall results of Table 3 support the conclusion that the uncertainty of this technique is small enough to validate the proposed procedure as another method for estimating the roughness of a surface. Nevertheless, there is high potential for improving the precision and stability of the measurement procedure. The experimental results shown in this article serve as a proof of principle, and several error sources have not yet been eliminated.

The most significant error sources are the implemented LCSLM and the laser source. To improve the accuracy of the method, a laser source with high intensity stability is mandatory. The key element of the design of this method is the LCSLM, so the quality of the results is directly linked to the functionality of this device. As discussed before, the used Holoeye PLUTO-VIS modulator relies on PWM scheme for digital addressing of the liquid crystals. One well-known problem with PWM schemes is that they cause the liquid crystals to flicker in a certain grey value range, which cannot be completely eliminated by external triggering [28]. The magnitude of phase flickering is connected to the selected digital addressing sequence and can be decreased by the application of other digital addressing sequences such as “5-5” or “0-6”. These schemes not only lead to lower temporal phase fluctuations but also to the reduction of the modulation range and the number of addressable phase levels [29]. To enhance the measurement process without loss of the modulation range, it is recommended to implement a LCSLM that applies analogue voltages to realise different phase shift levels (e.g., manufactured by Hamamatsu or Meadowlark).

Last but not least, the reflectivity of the targets has to be considered. In the presented experiments, three targets with reflection coefficients almost equal to the estimation of $V_{\mathrm{norm}}$ are investigated. To extend the technique to other materials, the influence of the reflectivity has to be taken into account in the theoretical approach.

Future studies could observe a 2-dimensional data analysis procedure in order to generate a map of the surface roughnesses.

## Figures and Tables

**Table 1:** Results for $R_{q,o}$ based on the modified simulation of roughnesses $R_{q,r}$.

**Modified Simulation Input**	10 nm	20 nm	40 nm	80 nm
**Modified Simulation Result**	10.0 ± 0.7 nm	20.0 ± 0.9 nm	39.9 ± 1.2 nm	77.6 ± 4.1 nm

**Table 2:** Experimental results of $R_{q,r}$ and $R_{a,r}$ for three targets measured by atomic force microscopy (AFM), stylus profiling, and the LCSLM with $\lambda_{1}=457.9\;nm$ and $\lambda_{2}=501.7\;nm$.

AFM		Target 1 (R_q_/R_a_)	Target 2 (R_q_/R_a_)	Target 3 (R_q_/R_a_)
		45.9/36.7 nm	12.3/9.8 nm	27.4/21.9 nm ^1^
stylus profiler	MP1	35.5/28.4 nm	15.7/12.6 nm	29.0/23.2 nm ^1^
	MP2	39.3/31.4 nm	17.0/13.6 nm	43.4/37.7 nm ^2^
LCSLM—$\lambda_{1}$	MP1	38.2/30.6 nm	16.8/13.4 nm	43.8/35.0 nm ^2^
	MP2	32.1/25.7 nm	16.2/13.0 nm	42.4/33.9 nm ^2^
LCSLM—$\lambda_{2}$	MP1	33.3/26.6 nm	12.0/9.6 nm	44.2/35.4 nm ^2^
	MP2	35.9/28.7 nm	16.9/13.5 nm	44.2/35.4 nm ^2^

^1^ untreated area of Target 3, ^2^ roughened area of Target 3.

**Table 3:** Uncertainty $U(\sigma_{o}=R_{q,o})$ of the experimental results in %.

		Target 1	Target 2	Target 3
LCSLM—$\lambda_{1}$	MP1	±0.9	±1.0	±0.4
	MP2	±0.3	±1.7	±0.9
LCSLM—$\lambda_{2}$	MP1	±0.6	±21.1	±0.5
	MP2	±1.0	±2.6	±0.7

## References

[1] Salazar F., Barrientos A. (2013). Surface roughness measurement on a wing aircraft by speckle correlation. Sensors.

[2] Chang C.-H., Kryder M.H. (1994). Effect of substrate roughness on microstructure, uniaxial anisotropy, and coercivity of Co/Pt multilayer thin films. J. Appl. Phys..

[3] Luo Z., Lu Y., Singer D.W., Berck M.E., Somers L.A., Goldsmith B.R., Johnson A.T.C. (2011). Effect of Substrate Roughness and Feedstock Concentration on Growth of Wafer-Scale Graphene at Atmospheric Pressure. Chem. Mater..

[4] Salazar Bloise F., Aulbach L., Jakobi M., Koch A.W. (2014). Rauheitsmessung an pharmazeutischen Tabletten mittels Angularer Speckle-Korrelation. TM Technisches Messen.

[5] Duparré A., Ferre-Borrull J., Gliech S., Notni G., Steinert J., Bennett J.M. (2002). Surface characterization techniques for determining the root-mean-square roughness and power spectral densities of optical components. Appl. Opt..

[6] Werth N., Salazar-Bloise F., Koch A. (2014). Influence of roughness in the phase-shifting speckle method: An experimental study with applications. Rev. Sci. Instrum..

[7] Thomas T.R. (1999). Rough Surfaces.

[8] Wyant J.C., Koliopoulos C.L., Bhushan B., George O.E. (2008). An Optical Profilometer for Surface Characterization of Magnetic Media. ASLE Trans..

[9] Wyant J.C., Koliopoulos C.L., Bhushan B., Basila D. (1986). Development of a Three-Dimensional Noncontact Digital Optical Profiler. J. Tribol..

[10] Wyant J.C., Caulfield H.J. (2002). White light interferometry. Proceedings of the AeroSense 2002.

[11] Windecker R., Franz S., Tiziani H.J. (1999). Optical roughness measurements with fringe projection. Appl. Opt..

[12] Ruffing B. (1986). Application of speckle-correlation methods to surface-roughness measurement: A theoretical study. J. Opt. Soc. Am. A.

[13] Muramatsu M., Eiju T., Shirai T., Matsuda K. (1997). Application of a liquid crystal spatial light modulator on optical roughness measurements by a speckle correlation method using two refractive indices. Opt. Laser Technol..

[14] Brodmann R., Thurn G. (1986). Roughness measurement of ground, turned and shot-peened surfaces by the light scattering method. Wear.

[15] Bodendorfer T. (2014). Ebenheits- und Rauheitsmessung Mittels Speckle-Interferometrie. Zugl. Dissertation.

[16] Matsuda K., Ye B.Q., Fukuchi N., Okamoto H., Hara T. (2007). Holographic vibration measurements of rough surfaces using a LCSLM. Opt. Commun..

[17] Bodendorfer T., Restrepo R., Belenguer T., Koch A.W., Doval Á.F., Trillo C. (2012). Applications of spatial light modulators in speckle interferometry. Proceedings of the SPECKLE 2012: V International Conference on Speckle Metrology.

[18] Chandley P.J. (1979). Determination of the standard deviation of height on a rough surface using interference microscopy. Opt. Quantum Electron..

[19] Atkinson J.T., Lalor M.J. (1980). The effect of surface roughness on fringe visibility in optical interferometry. Opt. Lasers Eng..

[20] Chandley P.J., Welford W.T. (1975). A re-formulation of some results of P. Beckmann for scattering from rough surfaces. Opt. Quantum Electron..

[21] Koch A.W. (1998). Optische Meßtechnik an Technischen Oberflächen. Praxisorientierte Lasergestützte Verfahren zur Untersuchung Technischer Objekte Hinsichtlich Form, Oberflächenstruktur und Beschichtung; Mit 4 Tabellen und 418 Literaturstellen.

[22] Elias C.N., Oshida Y., Lima J.H.C., Muller C.A. (2008). Relationship between surface properties (roughness, wettability and morphology) of titanium and dental implant removal torque. J. Mech. Behav. Biomed. Mater..

[23] Werth N., Müller M.S., Meier J., Koch A.W. (2011). Diffraction errors in micromirror-array based wavefront generation. Opt. Commun..

[24] Aulbach L.M., Koch A.W., Mendoza Santoyo F., Mendez E.R. (2015). Generation of high-resolution dynamic wavefronts for speckle-based measurements of complex surface shapes. Proceedings of the SPECKLE 2015: VI International Conference on Speckle Metrology.

[25] Hermerschmidt A., Osten S., Krüger S., Blümel T., Cheriaux G., Hooker C.J., Stupka M. (2007). Wave front generation using a phase-only modulating liquid-crystal-based micro-display with HDTV resolution. Proceedings of the International Congress on Optics and Optoelectronics.

[26] Cheremkhin P.A., Evtikhiev N.N., Krasnov V.V., Rodin V.G., Starikov S.N., Bjelkhagen H.I., Bove V.M. (2014). Reduction of phase temporal fluctuations caused by digital voltage addressing in LC SLM “HoloEye PLUTO VIS” for holographic applications. Proceedings of the SPIE OPTO.

[27] Taylor J.R. (1997). An Introduction to Error Analysis. The Study of Uncertainties in Physical Measurements.

[28] Farre A., Shayegan M., Lopez-Quesada C., Blab G.A., Montes-Usategui M., Forde N.R., Martin-Badosa E. (2011). Positional stability of holographic optical traps. Opt. Express.

[29] Bondareva A.P., Cheremkhin P.A., Evtikhiev N.N., Krasnov V.V., Starikov R.S., Starikov S.N. (2014). Measurement of characteristics and phase modulation accuracy increase of LC SLM “HoloEye PLUTO VIS”. J. Phys. Conf. Ser..

